# Acute-on-chronic liver disease enhances phenylephrine-induced endothelial nitric oxide release in rat mesenteric resistance arteries through enhanced PKA, PI3K/AKT and cGMP signalling pathways

**DOI:** 10.1038/s41598-019-43513-y

**Published:** 2019-05-06

**Authors:** Laura Caracuel, Esther Sastre, Pablo Llévenes, Isabel Prieto, Tania Funes, Mª Ángeles Aller, Jaime Arias, Gloria Balfagón, Javier Blanco-Rivero

**Affiliations:** 1 Departamento de Fisiología, Facultad de Medicina, Universidad Autónoma de Madrid, Madrid, Spain; 2 Instituto de Investigación Hospital Universitario La Paz (IdIPaz), Madrid, Spain; 3 Departamento de Cirugía General y Digestiva, Hospital Universitario la Paz, Madrid, Spain; 4 Cátedra de Cirugía, Facultad de Medicina, Universidad Complutense de Madrid, Madrid, Spain; 5 Centro de Investigación Biomédica en Red (CIBER) de Enfermedades Cardiovasculares, Madrid, Spain

**Keywords:** Cardiovascular diseases, Liver cirrhosis

## Abstract

Acute-on-chronic liver disease is a clinical syndrome characterized by decompensated liver fibrosis, portal hypertension and splanchnic hyperdynamic circulation. We aimed to determine whether the alpha-1 agonist phenylephrine (Phe) facilitates endothelial nitric oxide (NO) release by mesenteric resistance arteries (MRA) in rats subjected to an experimental microsurgical obstructive liver cholestasis model (LC). Sham-operated (SO) and LC rats were maintained for eight postoperative weeks. Phe-induced vasoconstriction (in the presence/absence of the NO synthase –NOS- inhibitor L-NAME) and vasodilator response to NO donor DEA-NO were analysed. Phe-induced NO release was determined in the presence/absence of either H89 (protein kinase –PK- A inhibitor) or LY 294002 (PI3K inhibitor). PKA and PKG activities, alpha-1 adrenoceptor, endothelial NOS (eNOS), PI3K, AKT and soluble guanylate cyclase (sGC) subunit expressions, as well as eNOS and AKT phosphorylation, were determined. The results show that LC blunted Phe-induced vasoconstriction, and enhanced DEA-NO-induced vasodilation. L-NAME increased the Phe-induced contraction largely in LC animals. The Phe-induced NO release was greater in MRA from LC animals. Both H89 and LY 294002 reduced NO release in LC. Alpha-1 adrenoceptor, eNOS, PI3K and AKT expressions were unchanged, but sGC subunit expression, eNOS and AKT phosphorylation and the activities of PKA and PKG were higher in MRA from LC animals. In summary, these mechanisms may help maintaining splanchnic vasodilation and hypotension observed in decompensated LC.

## Introduction

Liver diseases are among the ten most frequent causes of death in the Western world^1^. In general, these pathologies are clinically characterised by jaundice, discoloured urine, pale stools, pruritus, spleen enlargement, collateral vessel development and portal hypertension, causing a high rate of morbidity and mortality in the human clinical field^1–3^. Rat experimental models of hepatic fibrosis resulting from obstructive cholestasis cause an inflammatory activation of hepatic stellate cells, which express different, sometimes overlapping, phenotypes during the course of the disease; initially they develop a functional contractile phenotype that is responsible for the triggering of portal hypertension. They can then transform themselves into fibroblasts, which synthetize and release collagen, consequently causing liver fibrosis, a portal blood flow obstruction, and thus enhancing portal hypertension. These cells also acquire an immunological function, which is characterised by the release of both cytokines and chemokines, and therefore attracts leukocytes and thus induces an inflammatory response by the neighbouring cells through a paracrine mechanism. Hepatic sinusoidal and Kupffer cells may also play relevant proinflammatory roles, by releasing multiple adhesion molecules and inflammatory mediators. This stimulates a hyperplasia of the biliary epithelium and induces a biliary proliferation that would also contribute to the development of portal hypertension^4,5^. Simultaneously to this increase in intrahepatic vascular resistance, the splanchnic bed vascular resistance begins to decrease, as an adaptive response to the intrahepatic haemodynamic alterations. The experimental models of liver cholestasis have shown decompensation within six weeks of surgery, together with hepatic encephalopathy and ascites, leading to acute-on-chronic liver failure. This decompensation can aggravate the cardiovascular disturbances, and cause hypotension and decreased effective blood volume, as well as increased cardiac output^6,7^, eventually leading to patient death.

Different mechanisms have been suggested as contributors to mesenteric vasodilation in liver diseases. Enhanced levels of vasodilator factors including endothelial nitric oxide (NO) and the cyclooxygenase derivate prostaglandin I_2_ (PGI_2_), as well as of adenosine, glucagon and atrial natriuretic peptide, have been reported^8–10^. Additionally, the response to vasoconstrictors like alpha adrenoceptor agonist noradrenaline, angiotensin II, thromboxane A_2_ (TXA_2_) or arginine-vasopressin have also been described as reduced^11–13^.

NO generation can be triggered by vasoconstriction in some vessels, as a consequence of sympathetic nerve discharge^14^ or by activation through the alpha1-adrenergic receptor agonist, phenylephrine (Phe)^15,16^. Dora *et al*.^15^ were the first to show that Phe led to an increase in endothelial cell calcium concentration that triggered NO release and consequently attenuated vasoconstriction. In line with this, stimulation of smooth muscle alpha1-adrenergic receptors also leads to endothelial NO synthase (eNOS) phosphorylation in mouse mesenteric arteries^17^ through complex mechanisms that include phosphorylation on ser1177. eNOS phosphorylation can be produced as a result of different enzymatic pathways, including AMPK, PKA, CaMKII or PI3K/AKT^18–27^. The PKA and PI3K/AKT signalling pathways are both reported to be enhanced in liver pathologies^28–31^.

In view of these results, we aimed to determine whether activating alpha-1 adrenoceptors with Phe facilitates the release of endothelial NO in MRA from rats subjected to microsurgical liver cholestasis (LC), a model of acute-on-chronic liver disease, as well as the possible enzymatic pathways implicated.

## Materials and Methods

### Animals

Male Wistar rats were obtained and housed in the Animal Facility of the Universidad Autónoma de Madrid (Registration number EX-021U). The research conforms to the European Commission Directive 86/609 CEE Art. 21 (1995) and the Guide for the Care and Use of Laboratory Animals published by the US National Institutes of Health (NIH Publication No. 85–23, revised 1996). This study has been approved by the ethical committee of the Universidad Autónoma de Madrid.

### Surgical procedure

Rats (Initial weight: 294.5 ± 2.9 g) were divided into two groups: Sham-operated (SO; n = 25), in which the common bile duct was only dissected; and microsurgical liver cholestasis (LC; n = 25), in which the extrahepatic biliary tract was microsurgically resected^7,32,33^. Surgery was performed under aseptic but not sterile conditions, using a binocular operatory microscope (Zeiss, OPMI 1-FR). Briefly, rats were anaesthetised with ketamine hydrochloride (100 mg/kg) and xylazine (12 mg/kg) i.m. Bile duct anatomy in the rat has an intrahepatic and an extrahepatic portion, similar to humans. However, the difference lies in that rats lack a gall bladder. Additionally, rat liver has four lobes, while human liver only has two. Therefore, the extrahepatic portion is comprised for four biliary ducts, one draining each lobe, all of which gather to form the common bile duct^4,5^. In the SO group, we identified and dissected the biliary ducts in continuity with the common bile duct up to the beginning of its intrapancreatic portion. This extrahepatic bile duct was not resected. In the LC group, the common bile duct was ligated (silk 4/0) and sectioned close to the beginning of its intrapancreatic portion. The dissection and excision of the bile ducts from the four liver lobes of the rat must be done without injuring either the portal or, and most importantly, the arterial vascularisation of these lobes. The abdomen was closed in two layers by continuous running sutures using an absorbable suture (3/0 polyglycolic acid) and silk (3/0). Buprenorphine s.c. (0.05 mg/kg/8 hours) was administered postoperatively for analgesia during the first 24 hours post-surgery.

Rats were housed at a constant room temperature, humidity and 12 h light/dark cycle with free access to tap water and standard rat chow. Systolic blood pressure (SBP) was measured using the tail-cuff method^7,34,35^ 8 weeks after surgery was performed.

### Portal vein pressure measurement

Portal vein pressure measurement (PP) was performed under anaesthesia (100 mg/kg ketamine hydrochloride, 12 mg/Kg xylazine, i.m.). Splenic pulp pressure, an indirect measurement of portal pressure (PP) was measured by inserting a fluid filled 20-gauge needle into the splenic parenchyma^7,36,37^. The needle was joined to a PE-50 tube and then connected to a pressure recorder (PowerLab 200 ML 201) and a transducer (Sensonor SN-844) with a Chart V 4.0 computer program (ADI Instruments); these were calibrated before each experiment. The pressure reading was considered satisfactory when a stable recording was produced. Previous studies have demonstrated the excellent correlation between splenic pulp pressure and PP^38^.

Afterwards, animals were sacrificed by exsanguination by puncture of the infrahepatic inferior vena cava. Ascitic liquid was collected, and liver, spleen and the mesenteric arcade were removed and placed in cold Krebs−Henseleit solution (KHS, in mmol/L: NaCl 115; CaCl_2_ 2.5; KCl 4.6; KH_2_PO_4_ 1.2; MgSO_4_∙7H_2_O 1.2; NaHCO_3_ 25; glucose 11.1, Na_2_EDTA 0.03) at 4 °C.

### Serum biochemical test

Blood samples were kept at room temperature for 2 hours, and afterwards centrifuged (2000 g, 10 min, 4 °C). The supernatant (serum) was collected and kept at −70 °C until use. Serum levels of the following hepatobiliary metabolites were determined in an autoanalyzer: total and direct bilirubin (TB and DB); alkaline phosphatase (AP); bile acids (BA); aspartate aminotransferase (AST); alanine aminotransferase (ALT); lactate dehydrogenase (LDH); total proteins (TP) and albumin,.

### Vessel preparation

For reactivity experiments the third order branch of the mesenteric arcade (diameter, in μm: SO: 263.25 ± 4.06, n = 10, LC: 268.04 ± 5.46, n = 10, P > 0.05) was dissected from the mesenteric bed, cleaned of connective tissue and cut into segments of approximately 2 mm in length. Two tungsten wires (40 µm diameter) were introduced through the lumen of the segments and mounted in a small vessel myograph (Danish Myo Technology A/S, Aarhus, Denmark) to measure isometric tension according to the method described by Mulvany and Halpern^39^. After a 30 min equilibration period in oxygenated KHS at 37 °C and pH 7.4, segments were stretched to their optimal lumen diameter for active tension development. This was determined based on the internal circumference-wall tension ratio of the segments by setting their internal circumference, L_0_, to 90% of what the vessels would have if they were exposed to a passive tension equivalent to that produced by a transmural pressure of 100 mmHg^39^.

### Experimental protocols

After a 45 minute-equilibration period, each arterial segment was exposed twice to KCl (120 mmol/L) to assess its maximum contractility. KCl exposure was maintained for 30 minutes, in order to get the maximal contraction produced by this agent. Then, the vessels underwent several washout periods until basal tone was recovered. Afterwards, the rings were contracted with a Phe concentration that induced approximately 50% of the KCl contraction, and then acetylcholine (ACh; 1 µmol/L) was added to assess the integrity of the endothelium. Some experiments were performed in endothelium-denuded vessels. The endothelium was removed before mounting the segments in the myograph by gently rubbing the intimal surface with a human hair. The effectiveness of endothelium removal was confirmed by the inability of ACh to relax Phe-contracted arteries.

After 60 minutes, cumulative concentration-response curves for Phe (100 nmol/L-0.1 mmol/L) were performed in arteries from both experimental groups. The effects of endothelium denudation, as well as of the nonselective NO synthase inhibitor N^ω^-nitro-L-arginine methyl ester (L-NAME, 100 µmol/L) were investigated on concentration-response curves for Phe.

The vasodilation induced by concentration-response curves for ACh (1 nmol/L-10 μmol/L), and NO donor diethylamine NONOate (DEA-NO, 0.1 nmol/L-0.1 mmol/L) was analysed in Phe-precontracted MRA from both SO and LC rats. The effect of the superoxide dismutase mimetic 4-hydroxi-2,2,6,6-tetramethylpiperidinoxyl (Tempol, 0.1 mmol/L) on DEA-NO vasodilator response was also determined. The vasoconstrictor response induced by TXA_2_ receptor agonist U46619 (0.1 nmol/L-5 μmol/L) was examined as well.

All drugs were added 30 min before performing the concentration-response curve, and did not alter the arterial basal tone.

### NO release

NO release was determined as previously described^34^. The second, third and fourth branches of MRA from SO and LC rats were equilibrated for 30 min in HEPES buffer (in mmol/L: 119 NaCl, 20 HEPES, 4.6 KCl, 1 MgSO_4_.7H2O, 0.15 Na_2_HPO_4_.12H_2_O, 0.4 KH_2_PO4, 5 NaHCO_3_, 1.2 CaCl_2_.2H_2_O, 5.2 glucose, pH 7.4) at 37 °C (stabilisation period). Afterwards, arteries were incubated with the fluorescent probe 4,5-diaminofluorescein (2 µmol/L) for 45 min, and medium was collected to measure the unspecific DAF fluorescence. Once the organ bath was refilled, Phe was added cumulatively (100 nmol/L-0.1 mmol/L) at 2 min intervals. The medium was only collected at the end of the concentration-response curve to Phe. The fluorescence of the medium was measured at room temperature using a spectrofluorimeter (Fluoroskan Ascent, MTX Labsystems, Finland, FL WINLAB Software) with excitation wavelength set at 492 nm and emission wavelength at 515 nm. Some segments were preincubated with L-NAME (100 μmol/L), the specific inducible NOS (iNOS) inhibitor 1400 W (1 μmol/L), the PKA inhibitor H89 (1 μmol/L) or with the PI3K inhibitor LY294002 (10 μmol/L). Considering the unspecificity of DAF fluorescence^40,41^, the stimulated NO release was calculated by subtracting the L-NAME resistant fluorescence from the NO release evoked by Phe. Also, blank measurement samples were collected from the medium without mesenteric segments in order to subtract fluorescence background emission. The amount of NO released was expressed as arbitrary fluorescence units per milligram of tissue.

### PKA and PKG activity assays

The second, third and fourth branches of MRA from SO and LC rats were frozen in liquid nitrogen and stored at –70 °C. PKA and PKG activities were respectively determined using a PKA kinase activity assay kit (Abcam), and a CycLex® Cyclic GMP dependent protein Kinase Assay Kit (MBL International Corporation). The frozen arteries were homogenised in a lysis buffer containing 1 mmol/L sodium vanadate, 1% SDS and pH 7.4, 0.01 mol/L Tris-HCl and centrifuged at 12,000 g for 10 min at 4 °C. The supernatant was then collected and used for the assay. Assays were performed following the manufacturers’ protocols. Protein content was measured using a DC protein assay kit (BioRad). Results were expressed as Optical Density (OD) Units/μg protein.

### Western blot analysis

Western blot analysis was performed as previously described^34^. For these experiments, 20 μg protein were loaded in each lane. We used a monoclonal purified mouse anti-eNOS/NOS Type III antibody (1:2500; BD Biosciences), a rabbit polyclonal antibody against eNOS phosphorylated in Ser1177 (P-eNOS, 1:2000; Abcam), a mouse monoclonal antibody against iNOS (1:5000 dilution; Transduction Laboratories), a mouse monoclonal anti-soluble guanylate cyclase –alpha1(sGC-α1) subunit antibody (1:500; Santa Cruz), a mouse monoclonal anti-soluble guanylate cyclase –beta1 (sGC-β1) subunit antibody (1:500; Santa Cruz), a mouse monoclonal anti-alpha 1 adrenergic receptor antibody (1:1000; Abcam), a rabbit polyclonal anti-PI 3 Kinase p85 beta antibody (1:2000; Abcam), a rabbit polyclonal anti-pan-AKT antibody (1:500; Abcam), a rabbit polyclonal anti-pan-AKT (phospho T308) antibody (P-AKT, 1:500; Abcam), and a monoclonal anti-β-actin-peroxidase antibody (1:50000; Sigma-Aldrich). Appropriate positive controls (+C) were used for each analysis (see figure legends).

### TXB_2_ and 6-ketoPGF1_α_ releases

The TXA_2_ and PGI_2_ releases were determined by monitoring their stable metabolites using the commercial kits Thromboxane 2 ELISA Kit and 6-keto Prostaglandin F1α ELISA Kit (Cayman Chemical), respectively. For sample collecting, MRA arteries were pre-incubated for 30 min in 2 mL of KHS at 37 °C, continuously gassed with a 95% O_2_–5% CO_2_ mixture (stabilisation period). This was followed by two washout periods of 10 min in a bath of 0.2 mL of KHS after which the medium was collected to measure basal release. Afterwards, arteries were subjected to a Phe concentration curve (100 nmol/L-0.1 mmol/L) at 1 min intervals. The medium was only collected at the end of the concentration-response curve to Phe. Basal and Phe-induced samples were immediately frozen in liquid nitrogen and conserved at −70 °C until the assays were performed. All assays were carried out according to the manufacturer’s instructions. Results were expressed as pg prostanoid/mL mg tissue.

### Drugs

Phenylephrine hydrochloride, acetylcholine chloride, diethylamine NONOate, diethylammonium salt, N^ω^-nitro-L-arginine methyl ester, H89, LY 294002, sodium vanadate, SDS, Trizma-Base, and 4-hydroxi-2,2,6,6-tetramethylpiperidinoxyl (Tempol) were purchased from Sigma-Aldrich (Madrid, Spain). U46619 was purchased from Cayman Chemical (Michigan, USA). Stock solutions (10 mmol/L) of drugs were made in distilled water, except for Tempol, H89 and LY 294002, which were dissolved in dimethylsulfoxide (DMSO), and administered so that the maximum DMSO concentration of the medium was less than 0.001%. These solutions were kept at −20 °C and appropriate dilutions were made on the day of the experiment.

### Statistical analysis

Phe and U46619-contractile responses were expressed as a percentage of the maximum response produced by KCl. ACh and DEA-NO relaxation responses were expressed as a percentage of the previous tone elicited by Phe. All values are expressed as means ± S.E.M. of the number of animals used in each experiment. To determine differences in the effect of endothelium removal or L-NAME preincubation on the response to Phe, we analyzed the differences between areas under the curve (dAUC), expressed as the percentage of increase in that area produced by each procedure. These data, together with Emax and logEC50, were obtained after adjusting the concentration-response curves with a non-linear regression (variable slope), using Graph Pad Prism 6.0 Software. Statistical analysis compared the curves obtained between experimental groups, or the curves obtained in the presence of the different substances with the control curve by means of a repeated-measure two-way ANOVA followed by a Bonferroni post-hoc test using Graph Pad Prism 6.0 Software. For the dAUC, Emax and logEC50 data, NO and TXB_2_ release, PKA and PKG activities, and Western blot experiments, an unpaired Student’s t test was used. A P value < 0.05 was considered significant.

## Results

### Animal evolution

All LC animals showed jaundice and choluria. Paraoesophageal, splenorenal and pararectal collateral vessels developed in LC animals (Data not shown). Body weight gain was less in LC animals. Low systolic blood pressure, portal hypertension, spleen and liver hypertrophy, and ascitic fluid extravasation were also observed in LC animals (Table 1).

**Table 1:** Effect of microsurgical liver cholestasis (LC) on body weight (BW), body weight gain (BWG), systolic blood pressure (SBP), portal pressure (PP), liver weight-to-body weight ratio (LW/BW), spleen weight-to-body weight ratio (SW/BW) and ascitic liquid extravasation in Wistar rats.

	BW (g)	BWG (g)	SBP (mm Hg)	PP (mm Hg)	LW/BW (%)	SW/BW (%)	Ascitic liquid (mL)
SO	425.8 ± 5.4	46.7 ± 5.9	110.7 ± 6.8	8.9. ± 3.6	3.14 ± 0.24	0.28 ± 0.04	—
LC	300.1 ± 7.6*	22.6 ± 21*	99.5 ± 4.5*	19.8 ± 5.1*	5.87 ± 1.05*	0.99 ± 0.21*	9.1 ± 1.6

Results are expressed as means ± S.E.M. ^*^P < 0.05 versus SO. n = 20 animals each group.

Regarding hepatic metabolism, we found an increase in total and direct bilirubin (TB; DB), alkaline phosphatase (AP), bile acids (BA), and aspartate aminotransferase (AST) in serum from LC rats. In addition, serum lactate dehydrogenase, total protein and albumin concentrations were diminished due to LC (Table 2).

**Table 2:** Serum levels of total bilirubin (TB); direct bilirubin (DB), alkaline phosphatase (AP), bile acids (BA), aspartate aminotransferase (AST), alanine aminotransferase (ALT), lactate dehydrogenase (LDH), total proteins (TP) and albumin (Alb) in Sham-Operated (SO) and microsurgical liver cholestasis (LC) rats.

	TB (mg/dL)	DB (mg/dL)	AP (U/L)	BA (µmol/L)	AST (U/L)	ALT (U/L)	LDH (U/L)	TP (g/dL)	Alb (g/dL)
SO	0.09 ± 0.01	0.001 ± 0.0002	112.34 ± 8.81	6.78 ± 0.75	146.66 ± 19.96	53.50 ± 7.68	1013.00 ± 128.53	5.97 ± 0.09	3′02 ± 0.04
LC	8.17 ± 0.39*	4.92 ± 0.22*	284.88 ± 15.49*	42.84 ± 3.97*	245.07 ± 17.55*	27.32 ± 2.54*	967.76 ± 86.91	4.28 ± 0.21*	1′62 ± 0.09*

Results are expressed as means ± S.E.M. ^*^P < 0.05 versus SO. n = 15–20 animals each group.

These observations confirm the effectiveness of this surgery in producing an acute-on-chronic liver failure.

### Vasoconstrictor response to KCl

To check smooth muscle integrity, and possible differences regarding the vascular contractile machinery between the experimental groups, we subjected the MRA segments to a depolarising solution of KCl (120 mmol/L). The vasoconstrictor response to KCl was similar in endothelium-intact segments from both experimental groups, and endothelium removal did not alter KCl-induced vasoconstriction (Table 3).

**Table 3:** Effect of exposure to a depolarizing solution of KCl (120 mmol/L) in endothelium-intact (+E) and endothelium denuded (−E) mesenteric resistance segments from from sham-operated (SO) and microsurgical liver cholestasis (LC) rats.

	Contraction (mN)	
	+E	−E
SO	16.72 ± 0.92	13.75 ± 2.15
LC	15.78 ± 0.82	12.85 ± 1.96

Results are expressed as means ± S.E.M. n = 10 animals each group.

### Acetylcholine-induced vasodilation

**Figure 1:**
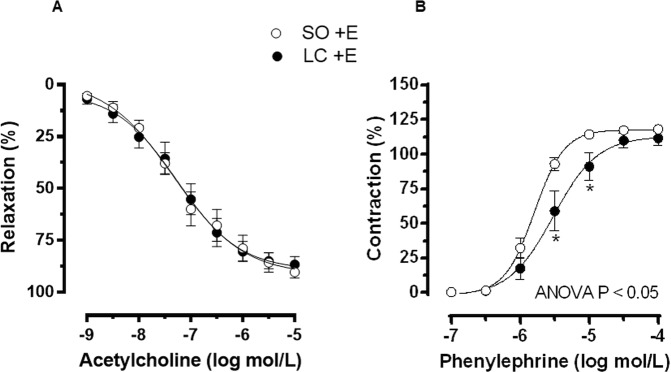
Effect of microsurgical liver cholestasis (LC) on the concentration-dependent relaxation to acetylcholine (**A**) and on the concentration-dependent contraction to phenylephrine (**B**) in endothelium-intact rat mesenteric resistance segments. Results (mean ± S.E.M.) were expressed as a percentage of the initial contraction elicited by phenylephrine (**A**) or by KCl (**B**). n = 10 animals in each experimental group. ^*^P < 0.05 (Bonferroni post-hoc test).

Endothelial function is generally studied by analysing the vasodilation induced by the endothelial agonist ACh. The vasodilator response to ACh in Phe-precontracted segments was similar in MRA from SO and LC rats (Fig. 1A, Table 4).

**Table 4:** Emax and log EC50 values of vasomotor responses to acetylcholine (ACh), phenylephrine (Phe), DEA-NO and U46619 in mesenteric resistance arteries from sham-operated (SO) and microsurgical liver cholestasis (LC) rats.

	SO		LC	
	Emax (%)	log EC50	Emax (%)	log EC50
ACh	89.28 ± 5.9	−7.25 ± 0.21	91.57 ± 6.4	−7.32 ± 0.19
Phe + E	117.8 ± 2.3	−5.79 ± 0.03	112.9 ± 7.2	−5.52 ± 0.09*
Phe –E	123.1 ± 2.1	−6.01 ± 0.03^#^	124.6 ± 3.3	−5.93 ± 0.04^#^
Phe + E + L-NAME	118.2 ± 5.4	−6.25 ± 0.13^+^	130.8 ± 7.3	−5.83 ± 0.10^+^
DEA-NO	91.09 ± 2.9	−6.45 ± 0.09	93.78 ± 3.2	−7.28 ± 0.13*
DEA-NO + Tempol	94.41 ± 2.9	−6.57 ± 0.08	91.99 ± 1.9	−7.55 ± 0.07*
U46619	111.2 ± 4.2	−8.01 ± 0.07	103.7 ± 5.0	−7.34 ± 0.08*

Results are expressed as means + S.E.M. n = 5–10 segments from different animals in each group. ^*^P < 0.05 SO vs. LC. ^#^P < 0.05 arteries with endothelium vs. arteries without endothelium. + P < 0.05 arteries without L-NAME vs. arteries incubated with L-NAME.

### Vasoconstrictor response to phenylephrine. Role of alpha-1 adrenoceptors

The concentration-dependent contractile response to the alpha-1 agonist Phe was lower in endothelium-intact MRA from LC compared to SO rats (Fig. 1B, Table 4).

**Figure 2:**
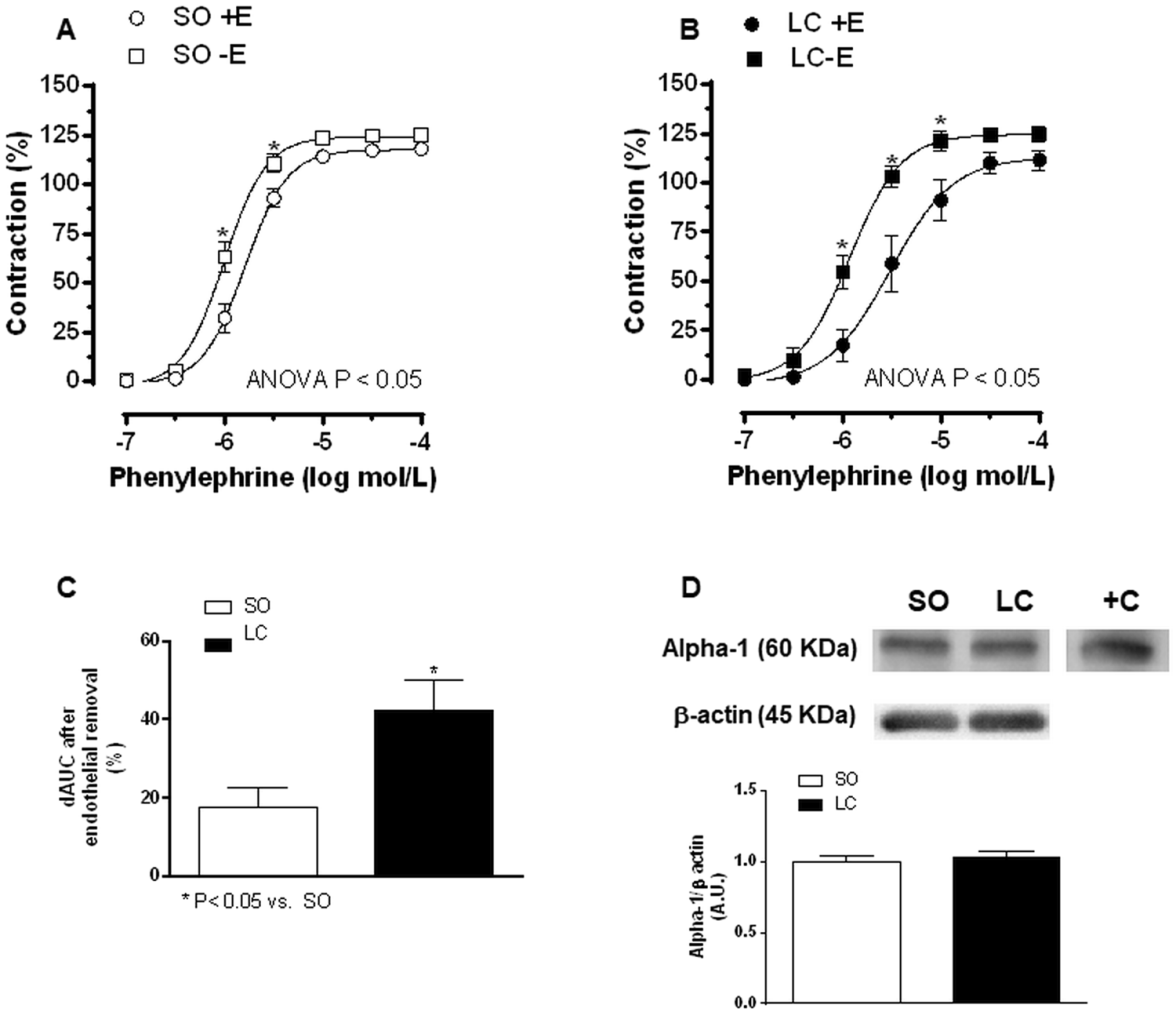
Effect of endothelial denudation on phenylephrine-induced vasoconstriction in mesenteric resistance arteries from Sham-Operated (SO, **A**) and microsurgical liver cholestasis (LC, **B**) rats. Results (mean ± S.E.M.) were expressed as a percentage of the initial contraction elicited by KCl. n = 10 animals per experimental group. ^*^P < 0.05 (Bonferroni post-hoc test). (**C**) Differences of area under the curve (in percentage) in the absence or presence of endothelium. (**D**) Western blot analysis for alpha-1 adrenoceptors in mesenteric resistance arteries from SO and LC rats. Each lane is representative of 8 isolated arterial segments from different animals in each group. A rat brain homogenate was used as a positive control (+C). Lower panel shows the densitometric analysis for the alpha-1 adrenoceptor expression. Results (mean ± S.E.M.) were expressed as the relation between the signal obtained for the protein analysed and the signal obtained for β-actin.

In order to determine whether these differences were due to alterations in endothelial factor release we eliminated the endothelium in several MRA segments from both SO and LC rats. We found that Phe-induced vasoconstriction was greater in both experimental groups after endothelial denudation. (Fig. 2A,B, Table 4). This increase was stronger in MRA from LC rats (Fig. 2C).

The last observation led us to hypothesise that LC did not alter the alpha-1 adrenoceptor signalling pathway. The fact that the alpha1-adrenoceptor expression was similar in segments from both animal groups confirmed this hypothesis (Fig. 2D).

### Role of endothelium-derived NO in the vasoconstrictor response to phenylephrine

**Figure 3:**
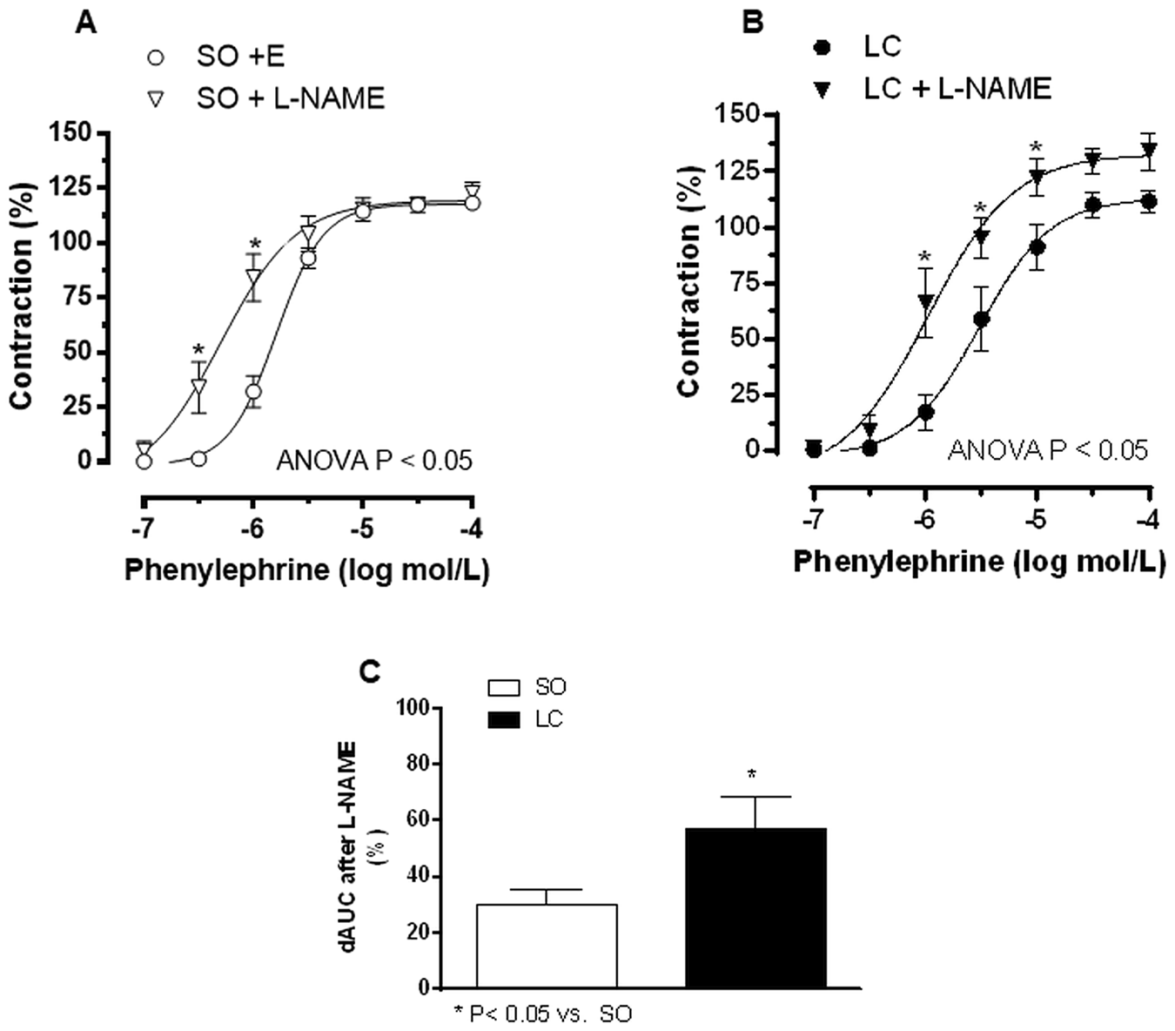
Effect of preincubation with the unspecific NOS inhibitor L-NAME (100 μmol/L) on the phenylephrine-induced vasoconstriction in mesenteric resistance arteries from Sham-Operated (SO, **A**) and microsurgical liver cholestasis (LC, **B**) rats. Results (mean ± S.E.M.) were expressed as a percentage of the initial contraction elicited by KCl. n = 10 animals per experimental group. ^*^P < 0.05 (Bonferroni post-hoc test). (**C**) Differences of area under the curve (in percentage) in the absence or presence of L-NAME.

To assess the contribution of endothelium-derived NO to the Phe-induced responses, segments were incubated with the NO synthase inhibitor L-NAME. This drug increased the response to Phe in arteries obtained from both experimental groups (Fig. 3A,B, Table 4). The increase was greater in LC than in SO animals (Fig. 3C). Additionally, the Emax and log EC50 values showed that the Phe-induced vasoconstriction in the presence of L-NAME was similar to the one observed in de-endothelised segments (Table 4).

**Figure 4:**
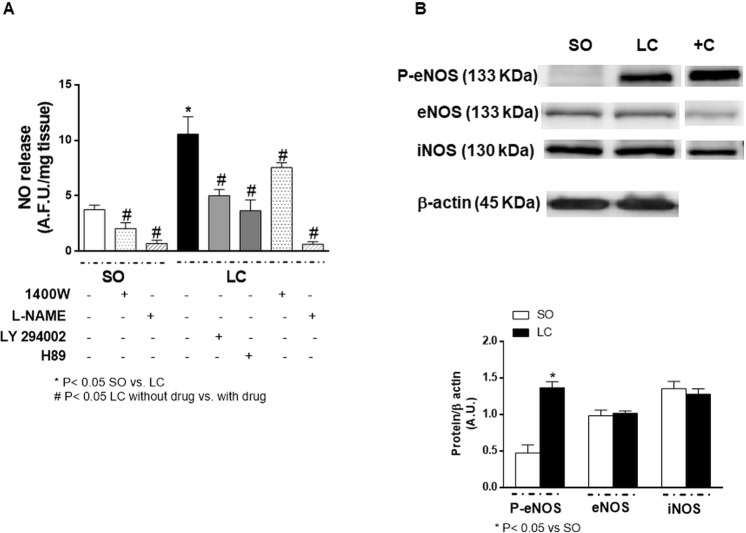
(**A**) Phenylephrine-induced NO release in mesenteric resistance arteries from sham-operated (SO) and microsurgical liver cholestasis (LC) rats. Influence of preincubation with the specific iNOS inhibitor 1400 W (1 μmol/L), the unspecific NOS inhibitor L-NAME (100 μmol/L), the PI3K inhibitor LY 294002 (10 μmol/L) and the PKA inhibitor H89 (1 μmol/L). Data were expressed as arbitrary fluorescence units/mg tissue. n = 6–16 segments from different animals in each group. (**B**) Western blot analysis for total and phosphorylated endothelial nitric oxide synthase (eNOS) in the Ser 1177 residue (P-eNOS) and inducible nitric oxide synthase (iNOS) in mesenteric resistance arteries from SO and LC rats. Each lane is representative of 8 isolated arterial segments from different animals in each group. Endothelial cells were used as a positive control (+C) for both eNOS and P-eNOS analysis. Activated macrophages were used as a positive control (+C) for iNOS analysis. Lower panel shows the densitometric analyses for the expression of each protein. Results (mean ± S.E.M.) were expressed as the relation between the signal obtained for the protein analysed and the signal obtained for β-actin.

Since the effect of L-NAME was different in segments from SO and LC rats, we aimed to determine the possible differences in endothelial NO release, observing that Phe induced NO release in mesenteric arterial segments from both groups. The increased NO release was greater in MRA from LC than in SO animals (Fig. 4A). Since both the PKA and PI3K/AKT signalling pathways may influence NO release, we used different inhibitors for these pathways (H89 and LY294002, respectively), to examine their possible implication in the enhanced NO release in MRA from LC rats. We found that both H89 and LY294002 decreased Phe-induced NO release in segments from LC rats. Preincubation with the specific iNOS inhibitor 1400 W diminished Phe-induced NO release similarly in segments form both SO and LC rats (in percentage of inhibition: SO: 42.8 ± 5.4; LC: 41.7 ± 3.5; P > 0.05; n = 6 animals each group), while L-NAME abolished Phe-induced NO release in both experimental groups (Fig. 4A). Since we found a low DAF fluorescence signal in both experimental groups (in arbitrary fluorescence units: SO: 0.71 ± 0.22; LC: 0.68 ± 0.21; P < 0.05; n = 6 segments from different animals each group), and considering the unspecificity of DAF fluorescence^40,41^, we subtracted the L-NAME resistant fluorescence from the NO release evoked by Phe in all the experimental conditions analysed.

**Figure 5:**
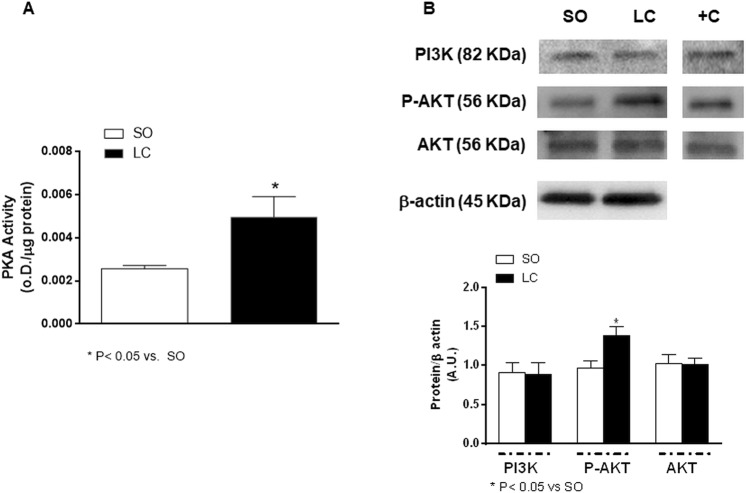
(**A**) Effect of microsurgical liver cholestasis (LC) on PKA activity in rat mesenteric resistance arteries. Results (means ± S.E.M.) are expressed in optical density (OD) units/μg protein. n = 8 animals each group. (**B**) Western blot analysis for PI3K and total and phosphorylated AKT in the T308 residue (P-AKT) in mesenteric resistance arteries from Sham-Operated (SO) and LC rats. Each lane is representative of 8 isolated arterial segments from different animals in each group. A rat brain homogenate was used as a positive control (+C). Lower panel shows densitometric analyses for the expression of each protein. Results (mean ± S.E.M.) were expressed as the relation between the signal obtained for the protein analysed and the signal obtained for β-actin.

The next step was to determine possible alterations in the expression/activity of the different enzymes implicated in the NO release. We saw that LC did not modify the expression of either iNOS or eNOS, but eNOS phosphorylation on Ser 1177 was increased (Fig. 4B). Furthermore, PKA activity was enhanced in MRA from LC rats (Fig. 5A), while PI3K expression was similar in segments from both groups, as was AKT expression. However, AKT phosphorylation in T308 residue was increased in MRA from LC rats (Fig. 5B). These results confirm an implication of both PKA and PI3K/AKT signalling pathways in the activation of eNOS and subsequent endothelial NO release in MRA from LC animals.

### Vasodilator response to exogenous DEA-NO

**Figure 6:**
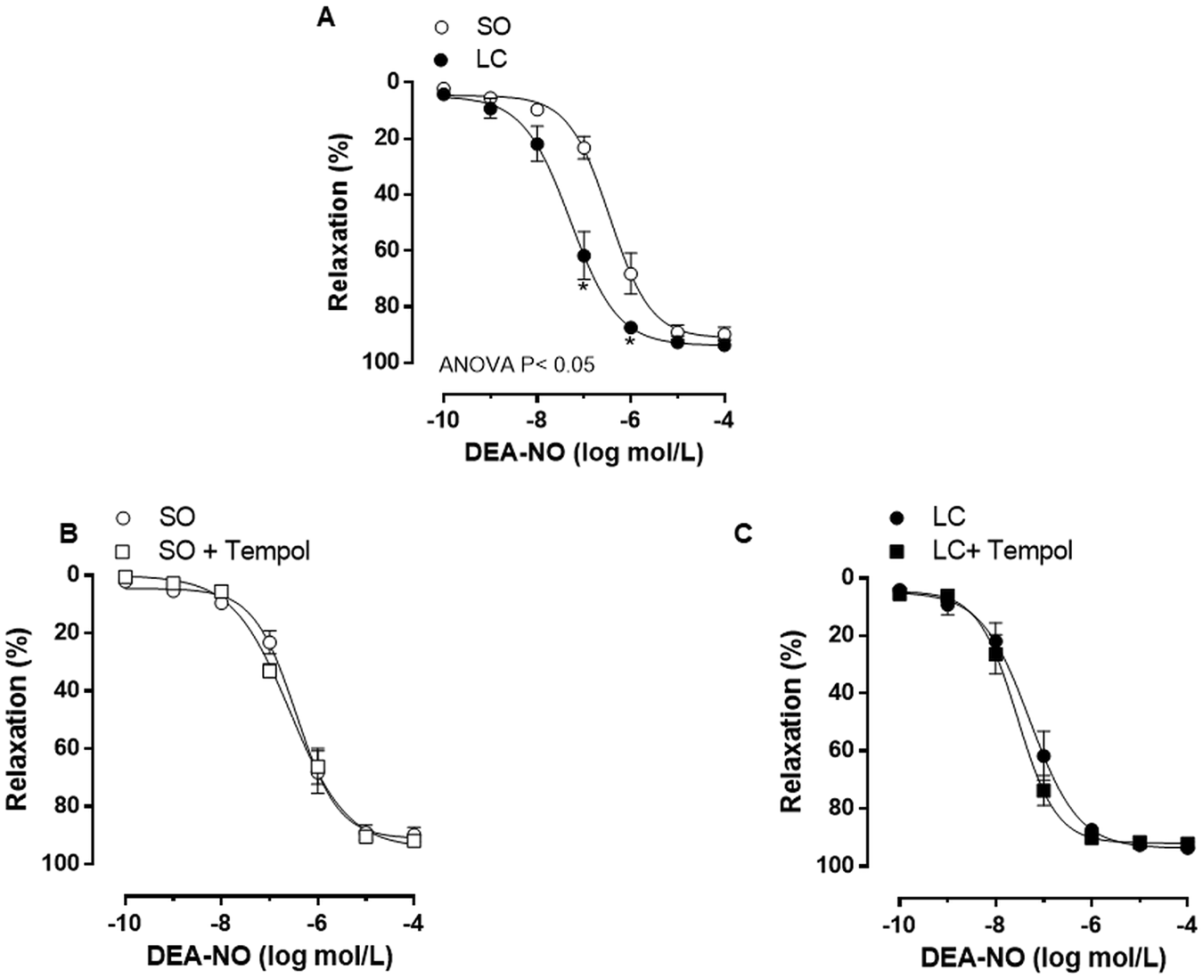
(**A**) Effect of microsurgical liver cholestasis (LC) on the concentration-dependent relaxation to NO donor DEA–NO in rat mesenteric resistance arteries. Influence of preincubation with the superoxide anion scavenger Tempol on mesenteric resistance arteries from Sham-Operated (SO, **B**) and LC rats. Results (mean ± S.E.M.) were expressed as a percentage of the initial contraction elicited by phenylephrine. n = 10 animals per experimental group. ^*^P < 0.05 (Bonferroni post-hoc test).

The differential NO role observed in our experimental conditions can also be produced by alterations in smooth muscle sensitivity to NO. When analysing the vasodilator response to NO donor DEA-NO we found a greater response in MRA from LC compared to SO animals (Fig. 6A, Table 4). Preincubation with the superoxide dismutase mimetic Tempol did not modify DEA-NO vasodilator response in either experimental group (Fig. 6B,C, Table 4), allowing us to rule out a possible influence of oxidative stress in these differences.

**Figure 7:**
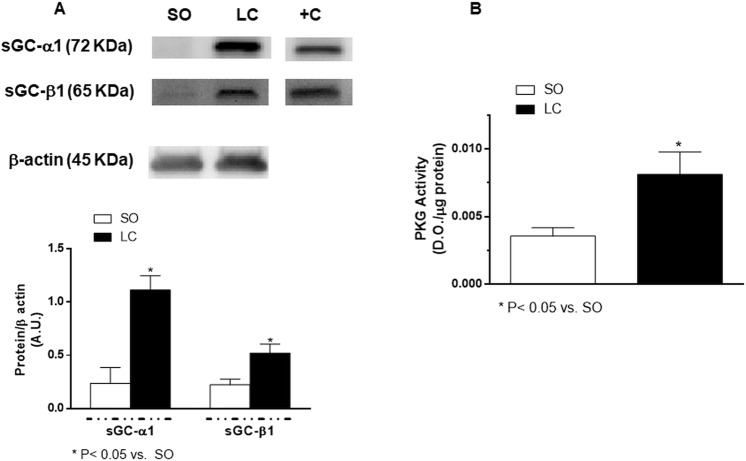
(**A**) Western blot analysis for sGC subunits sGCα-1 and sGCβ-1 in mesenteric resistance arteries from Sham-Operated (SO) and microsurgical liver cholestasis (LC) rats. Each lane is representative of 8 isolated arterial segments from different animals in each group. A rat brain homogenate was used as a positive control (+C). Lower panel shows densitometric analyses for the expression of each protein. Results (mean ± S.E.M.) were expressed as the relation between the signal obtained for the protein analysed and the signal obtained for β-actin. (**B**) Effect of LC on PKG activity in rat mesenteric resistance arteries. Results (means ± S.E.M.) are expressed in optical density (OD) units/μg protein. n = 7 animals per group.

Since cGMP plays a major role in the vasodilation induced by NO, we consequently analysed whether possible differences in the NO-cGMP signalling pathway could be implicated in the enhanced NO-induced vasodilation. We found that the expression of the soluble guanylate cyclase (sGC) subunits, sGCα-1 and sGCβ-1, was enhanced by LC (Fig. 7A). What is more, LC augmented PKG activity in MRA (Fig. 7B).

### Effect of LC on prostanoid participation

**Figure 8:**
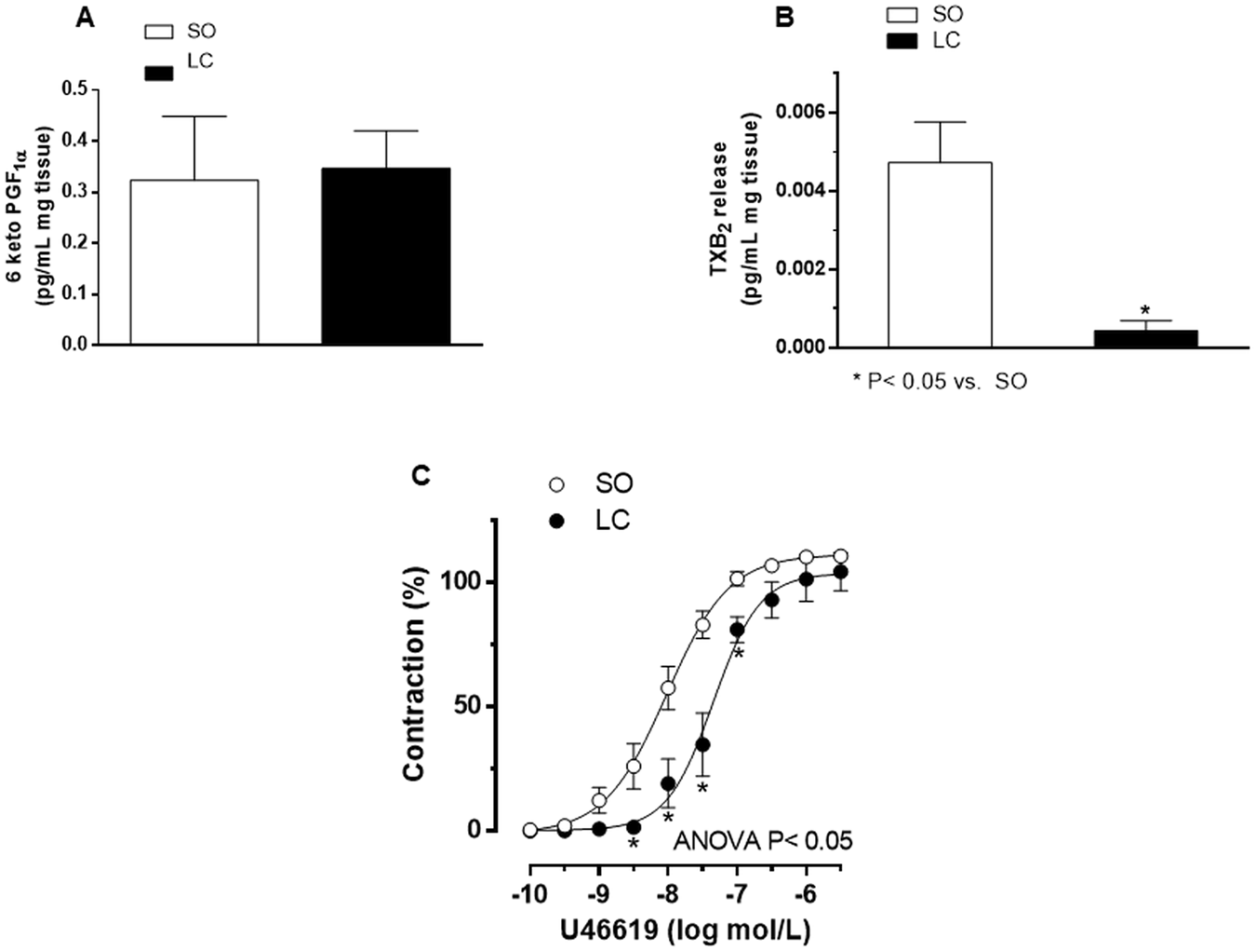
Effect of microsurgical liver cholestasis (LC) on 6-Keto PGF1_α_ release (**A**), TXB_2_ release (**B**). Results (mean ± S.E.M.) were expressed as pg prostanoid/mL mg tissue. (**C**) Vasoconstrictor-response to TXA_2_ receptor agonist U46619 in mesenteric resistance arteries from Sham-Operated (SO) and microsurgical liver cholestasis (LC) rats. Results (mean ± S.E.M.) were expressed as a percentage of the initial contraction elicited by KCl. n = 5–7 animals each experimental group. ^*^P < 0.05 (Bonferroni post-hoc test).

Aside from NO, the endothelium releases other vasoactive factors, such as prostanoids. The main vasoactive prostanoids observed in MRA are TXA_2_, and PGI_2_. When analysing the release of these prostanoids by monitoring their stable metabolites, we found that basal prostanoid release was similar in both experimental groups (TXB_2_, in pg/mL mg tissue: SO: 0.001 ± 0.0006; LC: 0.0009 ± 0.0003; P > 0.05; 6-keto PGF1_α_, in pg/mL mg tissue: SO: 0.003 ± 0.0007; LC: 0.002 ± 0.0005; P > 0.05). What is more, LC decreased Phe-induced TXB_2_ release in MRA from LC (Fig. 8A), while it did not modify 6-keto PGF1α release (Fig. 8B). These results indicate that the vasoconstrictor prostanoid TXA_2_ also has an important role in the diminished Phe-induced vasoconstriction observed in segments from LC rats. To confirm this, we examined the possibility of a possible differential vasoconstrictor effect of TXA_2_, and found that the vasoconstrictor response to TXA_2_ receptor agonist U46619 was diminished in MRA from LC animals (Fig. 8C, Table 4).

## Discussion

The main results of this study show a decreased vasoconstrictor response to Phe in MRA from LC rats as compared to control arteries. This hyporeactivity is due to NO overproduction caused by 1) augmented PKA and PI3K/AKT signalling pathway activity, 2) eNOS activation through phosphorylation at its Ser1177 residue, and 3) enhancement of the cGMP-signalling pathway activity.

Worldwide, liver pathologies with their associated comorbidities and fatalities are quite prevalent^1^. Among the multiple causes of liver pathology, one of the most characterised is liver cholestasis, which causes a high rate of morbidity and mortality in the clinical field^1–3^. The experimental models of rat hepatic fibrosis through obstructive cholestasis initially produce a functional contractile phenotype in hepatic stellate cells, which transform themselves into fibroblasts that release collagen and then proinflammatory cytokines, thus producing an obstruction of portal blood flow that leads to portal hypertension^4,5^. Clinically, the symptoms of these pathologies are jaundice, discoloured urine, pale stools, pruritus, enlarged spleen, collateral vessel development and portal hypertension, symptoms that may be clinically latent or mild in the first phases. Once the pathology becomes decompensated, all the above symptoms become aggravated, producing a clinical picture of acute-on chronic liver failure. In the present experimental study, liver fibrosis and hepatomegaly appeared in LC animals. Furthermore, there is a decrease in systolic blood pressure, accompanied by portal hypertension, while splenomegaly, collateral portosystemic circulation and ascitis were found in LC rats, as we reported earlier^7^. These modifications concur with those of decompensated liver cholestasis, being its evolution faster than the observed in the clinical field, hence making this experimental model appropriate for the translational study of the alterations associated with this disease.

The vascular disturbances in liver cholestasis have been the object of multiple studies. Mesenteric vasculature plays a key role in the development of the hyperdynamic circulatory syndrome of liver pathologies, which can be related either to increased response to vasodilator factors and/or a blunted vasoconstrictor response to different agonists. The participation of vasoactive factors is known to differ depending on the evolution of the pathology in other liver pathologies^12,34,36,42^. In line with this, we have previously reported increased sympathetic discharge as an attempt to counteract the marked splanchnic vasodilation that persists in rat mesenteric artery from decompensated liver cholestasis^7^. Although alterations in conductance vessels function have been reported in this condition^43,44^, to the best of our knowledge few studies have analysed the possible alterations of vasoconstrictor responses in MRA in the acute-on-chronic liver failure associated to decompensated liver cholestasis. It is widely known that the decreased MRA vascular tone in liver pathologies has several causes, including a diminished vasoconstrictor response to alpha-adrenergic agonists, a pivotal mechanism implicated in the development and maintenance of this hyperdynamic circulation. In line with this, our results showed a blunted vasoconstrictor response to alpha-1 agonist Phe in MRA from LC animals, and this decrease was similar to those reported in MRA from severely cirrhotic animals by different authors. Some reports agree with our observations^45–47^, but others have found no differences in Phe-induced vasoconstriction^48,49^. This result would indicate that the maintenance or alteration of Phe-induced vasoconstriction depends on the nature and/or the severity of the pathology.

The Phe blunted vasoconstriction observed in LC animals could be associated with alterations in the contractile machinery. However, the vasoconstrictor response after exposition to a depolarizing solution of KCl did not differ between segments from the two experimental groups, ruling out this possibility. This result agrees with ours and other’s previous reports describing the vascular effects of liver pathologies^7,34–37,50^. However, still other reports by other groups have shown a hyporeactivity to KCl in liver diseases^51,52^. Nevertheless, we must remember that the effects of liver pathologies can vary very widely, ranging from a mild liver cirrhosis to the acute-on-chronic liver failure described in the present study. The manners in which these pathologies are experimentally induced can also have different influences on the respective vascular beds. Thus, the evolution of the pathology, as well as the vascular bed used could explain the discrepant results in the different studies.

The vasoconstrictor response to Phe is mediated by the activation of post-synaptic alpha1 adrenoceptors, present in both endothelial and smooth muscle cells in this vascular bed^53–57^. In a previous study we have demonstrated that LC did not modify smooth muscle cell alpha1 adrenoceptor expression in mesenteric vasculature^7^. We also observed no differences in intact MRA in the present study. Regarding endothelial alpha-1 adrenoceptors, and using precontracted rat mesenteric resistance arteries, Filippi *et al*.^54^ demonstrated a weak increase in endothelial NO release after endothelial alpha 1 adrenoceptor stimulation with low concentrations of Phe. However, we and other authors had previously reported that the addition of low Phe concentrations in arteries did not alter basal tone^58–61^, and an initial contraction occurred only when micromolar concentrations of Phe were reached. These observations agree with a previous study by Dora *et al*.^61^, which reported that the increase in intracellular Ca^2+^ produced in endothelial cells from MRA after stimulation with Phe was minimal in basal conditions. Consequently, we consider that, if alterations in endothelial alpha 1 adrenoceptor function existed they would be quite minor and no relevant.

Of the numerous substances to be proposed as possible mediators in the decreased splanchnic resistance, endothelial factors are thought to play a major role. The vasodilator response to ACh is widely used to determine possible alterations in endothelial function. Increases^13,34,52,62^, decreases^63^ and no changes^64^ in ACh-induced vasodilation have been reported in mesenteric vasculature from different rat models of liver disease. These inconsistencies suggest that the alterations in ACh relaxation depend on the aetiology of the liver pathology, as well as on the vascular bed used. In our experimental conditions, we observed similar ACh-induced vasodilator responses in MRA from both control and LC rats, suggesting that endothelial function is not affected in our decompensated LC model. However, endothelium denudation enhanced the Phe-induced vasoconstriction in MRA from both experimental groups, and this increase was greater in LC arteries, suggesting that the endothelium plays a different modulatory role of the Phe response in SO and LC rats.

NO is one of the main factors involved in the blunted mesenteric resistance of liver diseases, and, consequently, it is partially responsible for the splanchnic vasodilatation in these patients. Phe stimulation of hamster arterioles has been reported to promote an increase in endothelial cell calcium concentration, thus triggering NO formation, which consequently attenuates arteriole constriction^15^. Similar results have been obtained in different vascular beds, including mouse and rat mesenteric arteries^52,65^. However, IP(3), rather than calcium, seems to have a role in vascular smooth muscle-to-endothelium communication after stimulation with Phe^65^. Consequently, an eNOS phosphorylation and subsequent increase in NO release occurs^17^. We observed that preincubation with the unspecific NOS inhibitor L-NAME increased Phe-induced vasoconstriction in MRA from both SO and LC rats, and the effect was greater in LC arteries. This result can be associated to the increase in endothelial NO release observed in this experimental group, an increase that could be associated to enhanced eNOS expression and/or activation. The lack of a difference in eNOS expression between SO and LC animals suggests that the enhanced NO release is due to augmented eNOS activity. Therefore, we studied the degree of phosphorylation on its Ser1177 residue, which we have previously reported to be responsible for eNOS activation in MRA^34^. This phosphorylation was greater in MRA from LC animals, confirming their higher eNOS activity and consequently explaining the increase in NO release observed in segments from LC rats. Aside from eNOS, iNOS has also been reported to be increased in both systemic and splanchnic vasculature in liver pathologies^34,66^. We observed that the specific iNOS inhibitor 1400 W diminished Phe-induced NO release to a similar extent in both SO and LC MRA segments. Additionally, no differences in iNOS expression were found in our experimental conditions. Both results allows us to rule out a different functional role for iNOS in our experimental procedure, and point out the possibility that this enhanced iNOS expression could be due to the similar surgical procedures induced in both SO and LC rats.

eNOS-Ser 1177 has been reported to be a target of several phosphorylation pathways, including both the PKA and PI3K/AKT signalling pathways^19–21,25–27^, the latter known to be present in liver^28–31^. Therefore, we aimed to determine possible alterations in the components and/or activity of these signalling pathways in LC. We found enhanced PKA activity in MRA from LC. Furthermore, the PI3K and AKT expressions were similar in MRA from both experimental groups while AKT phosphorylation was increased in arteries from LC rats. Taken together, these results suggest that hyperactivation of either the PKA and/or the PI3K/AKT signalling pathways could be responsible for an endothelial NO over-release in LC. The fact that the inhibition of either PKA or PI3K diminished Phe-induced NO release in arteries from LC rats supported us in confirming this hypothesis.

Apart from NO, other powerful vasoactive factors such as prostanoids, endothelial-derived contracting factors and endothelium-derived hyperpolarizing factor (EDHF) are released from the endothelium in these arteries^67^, and have a relevant role in regulating the vascular response to different agonists, such as Phe. Surprisingly, we observed that Phe-induced vasoconstriction in the presence of L-NAME in MRA from LC was similar to that observed in de-endothelized segments, indicating an exclusive role for NO after stimulation with Phe in these animals, and excluding the participation of the other endothelial factors. Similar results were also reported in aorta and mesenteric arteries from rats subjected to common bile duct ligation^68,69^. Multiple interactions have been reported between endothelial factors. Relevant results have been described by many authors, showing different mechanisms for TXA_2_ modulation of EDHF depending on the vascular bed analysed^70^, including an important role for prostanoids in situations where NO is enhanced^34,36^. TXA_2_ and PGI_2_ are the main prostanoids implicated in the maintenance of vascular tone. Increased TXA_2_ function has been described in hepatic vasculature in liver diseases^71,72^, with decreases reported in splanchnic vascular bed^34,73^, both results agreeing with the opposing alterations observed in these vasculatures. In our experimental conditions, we observed that the vasoconstrictor response to the TXA_2_ analogue U46619 was diminished in arteries from LC animals, showing that not only the vasoconstrictor response to Phe but also to other agonists is affected by this pathology. To sum up, TXA_2_ release was practically abolished in arteries from LC animals, as previously reported in both human and animal models^34,73^. This result confirms that the participation of this contractile prostanoid, if it exists, is very limited in mesenteric vascular bed in LC. On the other hand, we previously reported a major role for PGI_2_ in the development of mesenteric vasodilation in a compensated liver cirrhosis model induced by CCl_4_^34^. However, the PGI_2_ release was similar in mesenteric vasculature from SO and LC animals, ruling out a possible involvement by this vasodilating prostanoid in the development of splanchnic circulation, and suggesting a rearrangement in endothelial factor participation depending on the pathology. We and other authors have reached a similar conclusion regarding endothelial and nervous factors^7,35,36,42^. Regarding EDHF, several studies have reported that its participation is only pertinent after inhibition of endothelial NO, since otherwise NO would tonically inhibit EDHF participation, while increased EDHF activity have been described after endothelial NO inhibition^74–76^. Together, these results indicate very complex interactions between the different endothelial factors and that the interactions vary with the vascular tissue as well as the pathology under study.

The reduced EDHF participation previously reported in mesenteric arteries from rats subjected to common bile duct ligation could be associated to excessive oxidative stress^69^. In line with this, we have observed enhanced superoxide anion release in MRA from cirrhotic rats induced by CCl_4_^34^. Elevation in oxidative stress decreases NO bioavailability. Consequently, we aimed to analyse the vasodilator response to the NO donor DEA-NO in the presence of the superoxide anion scavenger, Tempol; this drug did not modify DEA-NO induced vasodilation in any experimental group, thus ruling out relevant oxidative stress effects in our experimental conditions. Nevertheless, DEA-NO induced vasodilation was greater in MRA from LC rats. NO has a very short half-life (20–30 s) and diffuses freely through the cellular membrane, acting mainly by activating the heterodimeric enzyme sGC, consequently enhancing cGMP production, and thus relaxing smooth muscle cells. Since we and other authors have observed increased implication of second messenger cGMP on endothelium-dependent and independent vasodilation in rat mesenteric vasculature in liver pathologies^13,37^, we aimed to determine whether the cGMP-signalling pathway would be enhanced in MRA from LC. For that purpose, we analysed the expression of sGC, and found increased levels of both the sGCα-1 and sGCβ-1 subunits. Similar results have been described in different tissues^77,78^, making us hypothesise that cGMP release may be increased in MRA from LC animals. cGMP is a second messenger that can induce, among other effects, the activation of PKG, an enzyme that plays a relevant role in vascular smooth muscle relaxation. The activity of this enzyme has been reported to be either altered or unaffected in different tissues from animals with liver pathologies^79–81^. We found greater PKG activity in MRA from LC animals, confirming a cGMP involvement in the enhanced vasodilator response to DEA-NO in our conditions and, consequently, participation by this second messenger in the hyporreactivity to Phe observed in this study.

As mentioned above, SBP was decreased in LC animals, as also described in patients with this pathology. Several mechanisms are implicated in the development and maintenance of this hypotension. First, the enhanced participation by the NO signalling pathway seems to be relevant in the hypotension observed in LC animals since it participates in the splanchnic vasodilation observed in this pathology and, consequently, produces a decrease in effective volaemia. In order to counteract this situation, the heart rate increases, raising cardiac output in these patients, who also develop collateral blood flow. However, we cannot forget that this pathology courses with a decrease in serum albumin, which is implicated in maintaining capillary colloid osmotic pressure. The decreased serum albumin results in ascitic liquid accumulation in the abdominal cavity, thus reducing total volaemia and, consequently, arterial pressure^82^. Working together, these mechanisms contribute to the hypotension characteristic of the pathology.

In summary, the main results of the present study shows a diminished vasoconstrictor response to Phe in MRA from decompensated LC rats. This hyporreactivity is a result of NO overproduction produced by augmented activation of both the PKA and the PI3K/AKT signalling pathways. Enhanced cGMP-signalling pathway activation could also be implicated in this response. These mechanisms can contribute to the maintenance of splanchnic vasodilation and hypotension observed in decompensated LC.
